# Over-expression of *EjLFY-1* Leads to an Early Flowering Habit in Strawberry (*Fragaria* × *ananassa*) and Its Asexual Progeny

**DOI:** 10.3389/fpls.2017.00496

**Published:** 2017-04-10

**Authors:** Yuexue Liu, Qian Zhao, Nan Meng, Huwei Song, Chaochao Li, Guibing Hu, Jincheng Wu, Shunquan Lin, Zhihong Zhang

**Affiliations:** ^1^College of Horticulture, Shenyang Agricultural UniversityShenyang, China; ^2^Jiangsu Key Laboratory for Eco-Agricultural Biotechnology Around Hongze Lake, Huaiyin Normal UniversityHuaian, China; ^3^Institute of Biotechnology in Horticultural Plants, South China Agricultural UniversityGuangzhou, China; ^4^Fujian Provincial Key Laboratory of Ecology-Toxicological Effects and Control Techniques of Emerging Contaminants, Putian UniversityPutian, China

**Keywords:** loquat (*Eriobotrya japonica*), *LEAFY*, transgenic strawberry, asexual progeny, flowering

## Abstract

As a master regulator involved in flower development, *LEAFY*-like gene has been demonstrated to play a key role in the flowering process regulation of angiosperms. Expression analysis of *EjLFY-1*, a *LEAFY* (*LFY*) homolog of loquat (*Eriobotrya japonica* Lindl.), indicated its participation in the regulation of flowering in loquat. To verify its function and potential value in the genetic engineering to shorten the juvenile phase, ectopic expression of *EjLFY-1* in strawberry (*Fragaria* × *ananassa*) was achieved using *Agrobacterium*-mediated gene transfer of a plant expression vector with the loquat *EjLFY-1* gene driven by the CaMV 35S promoter. Totally 59 plantlets were verified to be the transformants. The presence, expression and integration of *EjLFY-1* in the transformants were assessed by PCR, quantitative real-time PCR and Southern blot, respectively. Constitutive expression of *EjLFY-1* in strawberry accelerated the flowering process in strawberry with the shorten necessary period for flowering induction, development of flower and fruit set. While vegetative growth habits of the transformants in the first cropping season were consistent with the WT ones. Meanwhile, both the flowers and fruits of the transformants were also as same as those of the WT ones. Furthermore, the early-flowering habit was maintained in their asexual progeny, the runner plants. While with continuous asexual propagation, the clones showed a more strengthen early-flowering phenotype, such as the reduced vegetative growth and the abnormal floral organs in individual plantlets. These results demonstrated the function of this gene and at the same time provided us new insights into the utilization potential of such genes in the genetic engineering of perennial fruits.

## Introduction

Flowering is one of the most important events in the life cycle of plants. Flowering process of plant is affected by both endogenous factors, such as hereditary characters or plant hormones levels, and environmental conditions, such as temperature or day length. Flowering at right time accommodating to the seasonal and endogenous signals is vital for the reproductive success of all plants. The flowering time determination in plants is controlled by complicated gene networks.

In the past decades, a great deal of progress has been accomplished in understanding the molecular basis of the regulatory mechanisms underlying flowering time in plants, especially studies in the molecular regulatory networks of *Arabidopsis* provides us a better understanding about it. Numerous genes involved in the flowering regulation were demonstrated. In *Arabidopsis*, for example, it has been reported that at least 180 genes are implicated in flowering-time control (Fornara et al., 2010), regardless of these unknown or undetected genes. One possible explanation for so many genes are involved in flowering regulation is that this process is affected by various factors. Normally, there are several genetically defined pathways which affect the flowering: the vernalization pathway, the photoperiod pathway, the gibberellin pathway, the autonomous pathway and the ambient temperature pathway (Fornara et al., 2010; Srikanth and Schmid, 2011). Such signaling pathways responding to endogenous and environmental signals converge on key regulators, such as *FLOWERING LOCUS T* (*FT*), which then activate other floral homeotic genes. For plant, although the organ where developmental decision leading to flowering occurs is shoot apex, leaf is the organ which sensing environmental signals. In *Arabidopsis*, the mobile signal FT has been demonstrated to be the long-distance signal moved from leaf to shoot apex and then induce the flowering (Corbesier et al., 2007). Expression of *FT* is affected by such pathway signals and its protein can promote flowering together with the meristem-specific bZIP transcriptional factor FD (Abe et al., 2005; Wigge et al., 2005). At SAM (shoot apical meristem), the FT-FD complex promotes the expression of the floral meristem identity genes such as *LEAFY* (*LFY*).

*LEAFY* and its homologs in other plants encode a kind of unique transcription factors that assign the floral fate of meristems, being thought as a meristem identity gene which determines floral identity (Moyroud et al., 2010). In the *lfy* mutants of *Arabidopsis*, homeotic transformations with leaf-like structures replacing the floral organs took place (Weigel et al., 1992). Expression of *LFY* is also regulated by many pathway signals. For example, GA (Eriksson et al., 2006) and auxin (Li et al., 2013) regulate the *LFY* expression in *Arabidopsis* and thereby affect the flowering habits. The master role of *LFY* as a meristem identity gene is reflected partly in activating its downstream flowering regulation factors, such as the directly activating of the expression of *AP1*, whose upregulation stands for the initiation of flower formation (Mandel and Yanofsky, 1995; Wagner et al., 1999; William et al., 2004; Liu et al., 2007). Coincided with its master role in flowering progress, over-expression of *LFY* or its homologs usually leads to an early-flowering phenotype not only in herbaceous plants such as *Arabidopsis* (Weigel and Nilsson, 1995) and rice (He et al., 2000), but also in woody plants such as hybrid aspen (Rottmann et al., 2000).

Interestingly, such precocious flowering phenotypes also could be found in the fruit trees such as citrus over-expressing *AtLFY* (Peña et al., 2001). Fruit trees are perennials with a juvenile phase lasting for years in which they are not competent to flower, despite whether in inductive environmental conditions or not. The existing of juvenile phase in fruit trees has been suggested to be a restricting factor for their genetic improvement or breeding process: most economic phenotypes associated with fruits of the hybrids cannot be detected in this phase. Such work mentioned above make it possible to break the juvenile stage of fruit trees with the gene modify technique. Actually, there have been several reports associated with the over-expression of flowering-related genes in fruit trees so as to shorten their juvenile phase. Duan et al. (2010) showed the expression of *AtAP1* induced precocious flowering in transgenic kumquat. The juvenile period in apple has also been reduced with the silencing of the *MdTFL1* gene (Kotoda et al., 2006) or over-expressing of *FT* or *FT* homologous gene (Trankner et al., 2010; Bergonzi and Albani, 2011).

Nevertheless, in perennial fruit species, new questions arouse regarding the stability of integration and expression of foreign genes, there are few reports reporting the stability of transgene integration and expression in plants growing in the field over years (Peña and Séguin, 2001; Rai and Shekhawat, 2014).

Loquat (*Eriobotrya japonica* Lindl.) is an evergreen fruit tree native to China and cultivated mainly in tropical and subtropical regions. Fruits of loquat can be consumed fresh or processed for jam, juice, wine, syrup, or as candied fruits (Lin et al., 1999). As a kind of woody fruit, loquat also has a long juvenile phase, which impedes both productivity and breeding efficiency. Esumi et al. (2005) first reported the existence of *EjLFY-1*, a *LFY* homolog from loquat, but its expression and function were not clarified until now for the insufficient transgenic system in loquat.

**FIGURE 1:**
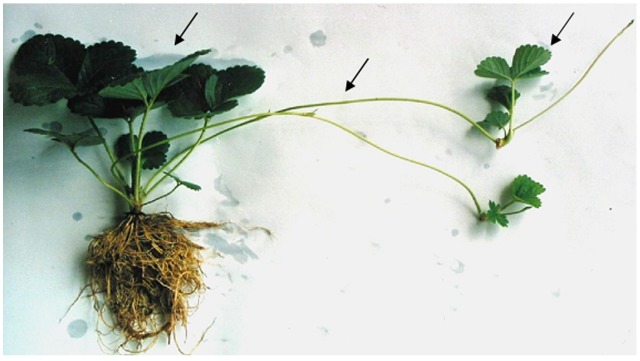
**Vegetative reproduction of strawberry by its stolon.** Arrows (from left to right) show the mother plant, stolon (runner) and the newly formed asexual plant.

Most gene function analysis in fruits were carried out with ectopic expression in model plants, such as *Arabidopsis* or tobacco, for their easier transformation and shorter life cycles. But one significant defect is that the characters related to fruits cannot be found in the transformants originated from such model plants. Strawberry has been considered as a good candidate for the function analysis of such genes. Strawberry owns short reproductive cycle and produces fruits. More importantly, strawberry shares similar gene sequence and genomic microcolinearity with other members of the Rosaceae family, including a large amount of the fruit trees such as apple, peach, pear, plum, apricot, cherry, and other species (Slovin et al., 2009). In addition to sexual reproduction, marked by flower and seed formation, strawberry can also reproduce asexually. It sends out stolon (also called runners), which makes new plants by producing roots and forming leaf clusters at some of the nodes where they touch the ground (**Figure 1**).

Without the gene’s recombination and segregation, propagation with stolon buds in strawberry makes it feasible to sufficiently investigate the transgenic effects of the exogenous genes in their asexual progeny, which is especially important for fruit trees. In production, fruit species must be propagated in asexual ways, such as grafting or layering, to maintain their characters stable. The stability of the transgenic effect in the asexual propagated progeny is crucial for the application of genetic engineering in fruit trees.

The main objective of the this study was to evaluate the function of *EjLFY-1* by investigating the ectopic expression effects of this gene on the flowering process in strawberry, and at the same time to investigate the stability of the effects of such transgene in the successive asexual propagated generations of the transformants.

## Materials and Methods

### Isolation of *EjLFY-1* Sequences

Gene-specific forward and reverse primers, EjLFYfwd and EjLFYrev (**Table 1**), were designed based on the reported *EjLFY-1* sequence (GenBank accession no. AB162033). Restriction enzyme sites were added additionally at the 5′-end and 3′-end of the primers for the subsequent vector construction. Flower bud and leaf of loquat cv. ‘Zaozhong No.6’ were used for the extraction of RNA and DNA, respectively. Full-length cDNA and DNA sequence of *EjLFY-1* were obtained by PCR. PCR products were cloned into pGEM-T easy vector (Promega, Madison, WI, USA) and then sequenced.

**Table 1:** Primers used in this study.

Name	Primers sequences (5′–3′)	Cyclic amplification methods	Expected amplification sizes
EjLFYfwd EjLFYrev	GGCTCTAGAATGGATCCAGATGCCTTC ACAGAGCTCTCAATAGGGCAGGTGCTCGC	32 cycles of 94° C for 1 min, 60°C for 1 min, 72°C for 1.5 min	1.2 Kb
DIGUP DIGDW	GTAGACGACAAGGACGACGA GATGGAGAGACGGGGATGAG	35 cycles of 94°C for 50 s, 53°C for 1 min, 72°C for 1 min	498 bp
KANF KANR	GTTCTTTTTGTCAAGACCGACC CAAGCTCTTCAGCAATATCACG	33 cycles of 94°C for 50 s, 61°C for 1 min, 72°C for 1 min	560 bp
KANUP KANDP	TCCATCAGCAACGTGTCGGTGCT GTGGAAAGGCGGTGAGCGATGAT	30 cycles of 94°C for 40 s, 51°C for 40 s, 72°C for 1 min	500 bp
AGRIF AGRIR	TCCATCAGCAACGTGTCGGTGCT GTGGAAAGGCGGTGAGCGATGAT	32 cycles of 94°C for 50 s, 59°C for 50 s, 72°C for 1 min	1.0 Kb
EjLFY-F	AGGGAGCACCCGTTCATT	40 cycles of 95°C for 10 s, 60°C for 20 s,	223 bp
EjLFY-R	GCATCTTCGGCTTGTTGA	72°C for 20 s	
FaAP1-F	AGAGGAAGGAGAAGGCAA	40 cycles of 95°C for 10 s, 60°C for 20 s,	230 bp
FaAP1-R	GAGAGTAAGGTCGAGCTGG	72°C for 20 s	
FaTFL1-F	GTCACTGCCAAACCGAGAG	40 cycles of 95°C for 10 s, 60°C for 20 s,	231 bp
FaTFL1-R	GAGAACAAACACAAACCTG	72°C for 20 s	


### Expression Analysis of *EjLFY-1* in the Flowering Process of Loquat

To determine the involvement of *EjLFY-1* in floral development of loquat, we detected its expression levels in different floral differentiation stages, which were generally grouped into four groups: vegetative buds, inflorescence buds, flower buds forming and blooming. Totally 12 samples (buds or flowers) were collected with 10-day intervals. 10 μg RNA from each sample was electrophoresed in 1.2% agarose gel and transferred to Hybond N^+^ nylon membranes (Roche, Swiss). A gene-specific fragment (498 bp) amplified by a pair of primers (DIGUP and DIGDW, **Table 1**) at the conserved region of its 3′ end was used as the probe for hybridization. The specific methods of probe labeling and hybridization are as described in the instruction offered by Roche DIG High Prime DNA labeling and Detection Starter Kit I (Roche, Swiss).

### Vector Construction and Strawberry Transformation

**FIGURE 2:**
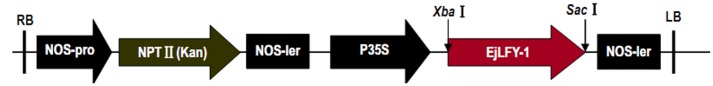
**Vector construction.** *EjLFY-1* was inserted in sense orientation between the *Xba* I and *Sac* I sites of the binary vector pBI121. RB Right border, P35S cauliflower mosaic virus 35S promoter, Nos, nos terminator; LB, left border.

Full-length cDNA sequence of *EjLFY-1* was subcloned into *Xba* I-*Sac* I sites of the binary vector pBI121 plasmid so that *EjLFY-1* was under the control of CaMV 35S promoter (**Figure 2**). Recombinant plasmid pBI121-35S::*EjLFY-1* was then introduced into the *Agrobacterium tumefaciens* strain EHA105.

Strawberry (*Fragaria* × *ananassa*) cv. Tudla was used for Agrotransfection. *A. tumefaciens*-mediated transformation was performed with the leaf-disk procedure (Nehra et al., 1990). Leaf segments were excised from *in vitro* plantlets grown on MS basal medium (Murashige and Skoog, 1962). The leaf segments were then cocultivated with *A. tumefaciens* harboring the plasmid pBI121-35S::*EjLFY-1*, followed by regeneration under the selection medium (MS+1.5 mg L^-1^ TDZ+0.4 mg L^-1^ IBA) containing kanamycin (30 mg L^-1^). Regenerated buds were excised and transferred to 1/2MS medium containing kanamycin (50 mg L^-1^) for rooting. Resistant plants from separate leaf explants were defined as independent transgenic events.

### Verification of Transgenic Plantlet

Genomic DNA were extracted from young leaves of kanamycin-resistant plants and the WT plants following a modified CTAB method (Ma et al., 2008), and then PCR analysis was carried out for detection of the insertion. Individual plants were tested for the presence of both *EjLFY-1* and *NPT II* genes separately. To avoid the potential pollution caused by *Agrobacterium* may exist in the regenerated resistant plants, the existing of *ChvA* gene (*Chromosomal virulence gene A*) which specially belongs to *Agrobacterium* was also checked and excluded by PCR. Primers (AGRIF and AGRIR) and amplification methods are listed in **Table 1**.

For further verification of the integration of *EjLFY-1*, Southern blotting was performed using a randomly selected plantlet which had been confirmed by PCR. About 20 μg of genomic DNA was digested with *EcoR* I and then electrophoresed on a 0.8% (W/V) agarose gel. The digested DNA in the gel was then transferred to an Immobilon-Ny^+^ membrane (Millipore, USA). A fragment of *NPT II* gene obtained by PCR (primers: KANF and KANR, **Table 1**) was labeled as the probe using the DIG DNA Labeling and Detection Kit II (Roche, Diagnostics, USA). Hybridization was carried out according to the manufacturer’s instructions.

Non-transgenic strawberry (WT) was used as the control, except that the *Agrobacterium* strain containing recombined plasmid was used as a control for detection of the existence of *ChvA* gene.

### Gene Expression Detection in the Transformants

Expression of *EjLFY-1* in the first generation transgenic plantlet was detected by RT-PCR. Plantlets grown in greenhouse were used to extract the total RNA using an improved CTAB method as described by Chang et al. (2007). Before precipitation, the RNA was treated with RNase-free DNase I (Takara Biotechnology Co., Dalian, China) at 37°C for 4 h to eliminate the eventual genomic contamination. RNA integrity was assessed on a 1.0% agarose gel, and concentration was estimated using a DU800 spectrophotometer (Beckman Coulter, Fullerton, CA, USA). The first-stand cDNA synthesis was performed with 1 μg total RNA and Oligo d(T)_18_ primer using Superscript II reverse transcriptase (Invitrogen, Carlsbad, CA, USA), according to the protocols provided by the manufacturers. Primers EjLFYfwd and EjLFYrev were used for the RT-PCR confirmation and 2 μl of the cDNA product was used as the template.

Quantitative real-time PCR (qRT-PCR) was performed to detect the expression of *EjLFY-1* in the third-generation propagated clones originated from stolon buds of the transformants. Leaf was used as the material for the detection of *EjLFY-1*, shoot apex meristem was used to examine the expression level of *FaAP1*, a homolog of *APETALA1* (*AP1*) in strawberry. Expression level of *FaTFL1*, homolog of the main floral repressor *TERMINAL FLOWER 1* (*TFL1*) in strawberry, was also detected. Gene-specific primers used in qRT-PCR were also listed in **Table 1**. RNA extraction and cDNA synthesis were carried out following the method mentioned above. The qRT-PCR was performed using SYBR Green qPCR Kit (Takara Biotechnology Co., Dalian, China) following the manufacturer’s instructions. qPCR was repeated with three biological replicates, and each sample was assayed in triplicate by PCR. The 26S rRNA gene of strawberry was used as a internal control.

### Field Trials

Independent transgenic lines and WT plants were rooted, transferred into pots and grown in a solar greenhouse in Shenyang Agricultural University (123°E, 41°N, Shenyang, Liaoning Province, China) at December of 2009. The agamic propagated plants from stolon buds of the transgenic plantlets were rooted and planted into new nutritional pots, and then separated from the donor plants.

At the end of each cropping seasons, old leaves and roots of the plantlets were partially removed and the plantlets were then transferred into a new nutritional pots separately.

### Horticultural Traits Analysis

The plantlets were successively flowering after growing in the greenhouse for 3–4 months and their horticultural traits were recorded individually. Flower timing of each sample was measured as the time period from transferring into pots to formation of the first flower. Plant height, flower numbers were also recorded.

### Data Analysis

The amino acid sequences were aligned and a phylogenetic tree was generated using the Clustal W method (Higgins et al., 1994). Boot strap values were derived from 1,000 replicate runs. The phylogenetic tree was constructed by MEGA 5.0 software (Tamura et al., 2011). Introns distribution diagram were drew with DNAMAN 6.0 software (Lynnon Biosoft, USA). The horticultural traits data were analyzed with EXCEL. Statistical analyses were performed using one-way ANOVA test, followed by Bonferroni’s test with SPSS software.

## Results

### Isolation and Sequence Characterization of *EjLFY-1*

**FIGURE 3:**
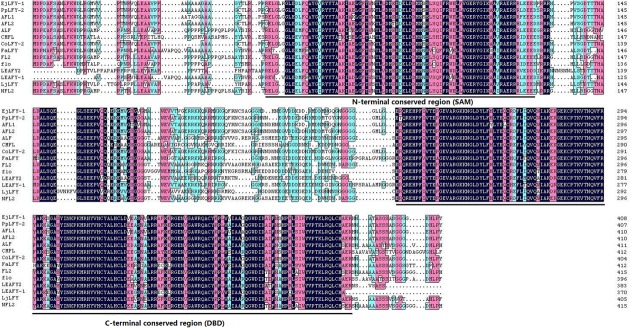
**Sequence alignment of EjLFY-1 and other LEAFY-like proteins.** Dashes indicate gaps to maximize the alignment. DBD motif at the C-terminal and shoot apical meristem (SAM) motif at the N-terminal are shown by lines on bottom of the alignment.

With a pair of gene-specific primers, a segment of 1,227 bp was isolated and sequenced. Sequence alignment showed that it is similar with the *EjLFY-1* sequence which reported by Esumi et al. (2005). There are only three nucleotide acids and one amino acid diverse between the isolated sequence and the reported one, which may be used as molecular tool for cultivar differentiation. Alignment of the amino acids sequences encoded by *EjLFY-1* and other *LFY*-like genes showed that they share the conserved domains with each other in the N-terminal and C-terminal, especially in the C-terminal conserve motif (**Figure 3**).

A phylogenetic tree was constructed by using the deduced protein of EjLFY-1 and the other reported LFY-like proteins with Neighbor-Joining Method (Supplementary Figure S1). EjLFY-1 falls in a small group constructed by other homologs of Rosaceae fruit trees, including the PpLFY of pear and the AFL1 and AFL2 of apple. These data are consistent with the traditional taxonomy, indicating their closely relationship during evolution process.

Full-length DNA sequence of *EjLFY-1* was also obtained. Sequencing result showed that g*EjLFY-1* (GenBank accession no. AY551183) was 2,602 bp in length, indicating the existing of intron(s). Alignment of g*EjLFY-1* with its cDNA sequence showed the existing of two introns. The first intron is 464 bp and the second one is 911 bp in length. The amount of introns in g*EjLFY-1* is also same as those reported *LFY*-like genes of other Rosaseae plants such as strawberry, dog rose and apple (Supplementary Figure S2).

### Expression of *EjLFY-1* in the Flowering Process of Loquat

**FIGURE 4:**
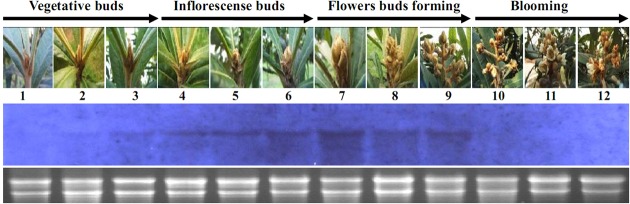
**Expression of *EjLFY-1* during the flowering process of loquat.** Materials were collected following the flowering development of loquat. For each lane, 10 μg of total RNA was loaded, blotted, and hybridized with an *EjLFY-1* probe. rRNA stained with ethidium bromide showing the equivalence of RNA loading between the samples.

Northern blotting data suggested that the transcription of *EjLFY-1* initiated in the buds that still in morphologic vegetative stages (**Figure 4**, lanes 2–3), except in the earliest detected vegetative bud (**Figure 4**, lane 1). Transcription level of *EjLFY-1* was increased gradually during the swelling period of the buds when the inflorescence meristems were initiated (**Figure 4**, lanes 4–6). Then the transcription level reached the topmost point when the inflorescence meristems started to generate flower meristems (**Figure 4**, lane 7). Once the flowers came into being, expression of *EjLFY-1* decreased (**Figure 4**, lanes 8–9). No transcripts were detected in little flowers or blooming flowers (**Figure 4**, lanes 10–12).

### Generating and Verification of Transformants

An over-expression construct with *EjLFY-1* CDS under the control of CaMV 35S promoter was introduced into strawberry cv. Tudla. Totally 59 resistant plantlets belonging to three independent lines (L1, L2, and L3) were got and rooted on MS medium containing kanamycin. The resistant plantlets and the WT ones were then transferred into pots and grown in greenhouse (Supplementary Figure S3).

**FIGURE 5:**
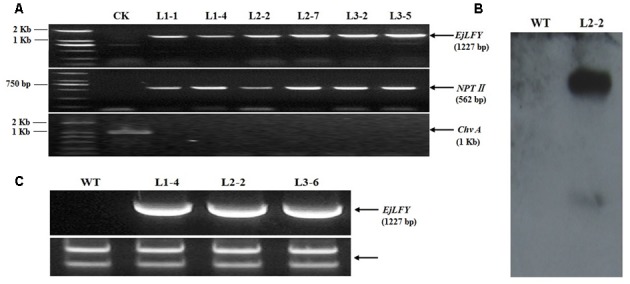
**Verification of the transformants. (A)** PCR detection for *EjLFY-1*, *NPT II* and *Chv A* genes, Marker: DL2000. **(B)** Southern blot analysis of L2-2. **(C)** Expression detection of *EjLFY-1* in random selected plantlets of three transgenic lines.

To verify the genomic integration of the target gene, presence of *EjLFY-1* and *NPT II* were confirmed by PCR separately. The expected specific fragments of both genes were detected in the randomly selected transformants (**Figure 5A**). While for the detection of *ChvA* gene, target fragment couldn’t be detected in any tested transformants (**Figure 5A**), indicating the detected fragments of *EjLFY-1* or *NPT II* were caused by transformation event, excluded the possibility of *Agrobacterium* continuation.

L2-2, a random selected PCR-positive transgenic plantlet, was further tested by using Southern blotting detection for the integration of *NPT II* gene. Southern hybridization with the *NPT II* probe showed that two bands were visible in L2-2 while none was observed in the WT control (**Figure 5B**). This result provides a further evidence of the *EjLFY-1* insertion in strawberry genotypes.

Expression level of *EjLFY-1* in independent transgenic line was detected by RT-PCR (primers: EjLFYfwd and EjLFYrev, **Table 1**). Results showed that transcripts of *EjLFY-1* could be detected in the tested transgenic lines (**Figure 5C**), but not in WT ones.

### Over-expression of *EjLFY-1* in Strawberry Promotes the Flowering Process

**FIGURE 6:**
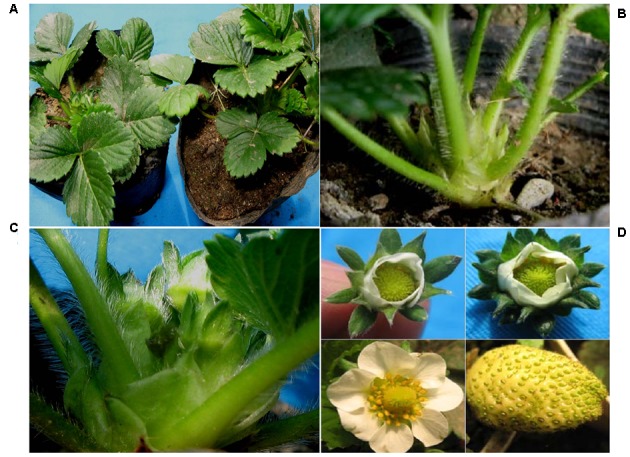
**Phenotypes of transformants and WT plants. (A)** WT (right) and transgenic (left) plants. **(C)** Close-up of the inflorescence formed in transformants, which was not seen in the WT ones **(B)**. **(D)** Morphology of the flowers and fruits of the transformants: Top left, flower of WT; top right, flower of one transgenic plantlet with doubled sepals; bottom, normal flower and fruit of transformants.

**FIGURE 7:**
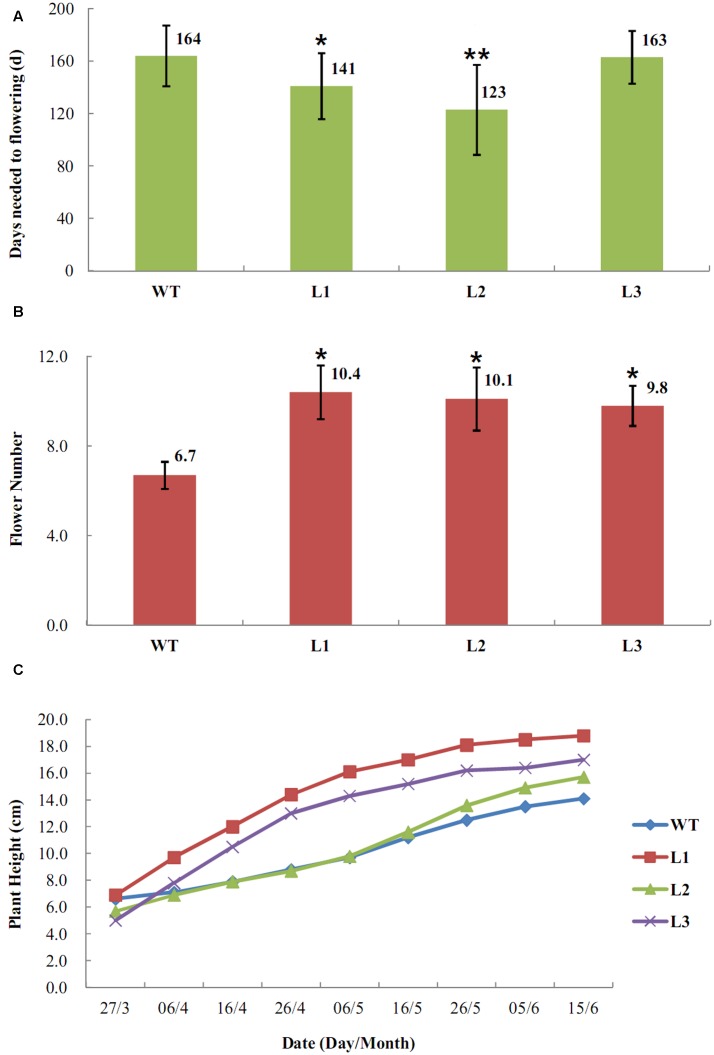
**Transgenic strawberry plants overexpressing *EjLFY-1* showed early-flowering habits. (A)** Average days needed for WT and transformants to flowering. *Asterisks* show significant difference comparing to WT (ANOVA, ^∗^*P* < 0.05, ^∗∗^*P* < 0.01). **(B)** Average flower numbers of the transformants and the WT ones. **(C)** Average plant height of the transformants and the WT ones.

To evaluate the effect of over-expression of *EjLFY-1* on the regulation of flowering process in strawberry, transgenic plantlets belonging to three independent lines (L1, L2, and L3) and the WT lines grown in small pots filled with nutrient soil were transferred into experimental greenhouse at December of 2009. It was found that plantlets belonging to L1 and L2 lines developed their inflorescences early and continuously at March of 2010, while the WT plants were still in vegetative growth at the meantime (**Figures 6A–C**). The early flowering habit of the transformants was very remarkable. Even at the April of 2010, when more than half plantlets of the L1 and L2 came into bloom, the WT ones didn’t develop inflorescences yet. Over-expression of *EjLFY-1* promoted flowering process of the plants belongs to L1 and L2, their average flowering time were 23 and 41 days shorter than that of the WT, respectively (**Figure 7**). While for plants of line L3, the change of flowering formation period was negligible in compare with WT.

### Other Horticultural Traits in Transgenic Strawberry Plants

Other horticultural traits of the transformants were also tested at 2010. Over-expression of *EjLFY-1* affected not only the flowering time of strawberry but also the flower quantities. Compared with WT plants, the transformants produced more flowers including the line L3 ones in which flowering time showed no significantly changes (**Figure 7B**). Even though, most flowers or fruits of the transformants showed no obviously changes compared with the WT ones, except that individual transgenic plantlets showing doubled sepals (**Figure 6D**).

Although over-expression of *EjLFY-1* promoted the flowering process of the transgenic lines, it didn’t reduce the vegetative growth habit of those transformants at the first growing season. For example, three transgenic lines even showed a little higher average height phenotype than the WT ones at each investigated point except the beginning (**Figure 7C**).

### Early-flowering Phenotype Could Be Maintained in Their Asexual Progeny

Almost all the fruit species in commercial production must be propagated by asexual ways so as to keep their stable traits. Normally the nursery stock of strawberry is runner plant which comes from stolon bud of the donor plant.

**FIGURE 8:**
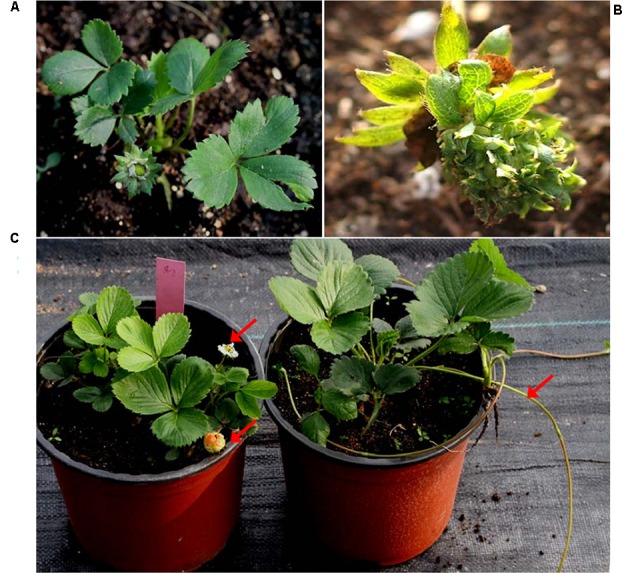
**More strengthen phenotypes of the progeny propagated from stolon buds of the transgenic strawberry. (A)** An very early flowering plant. **(B)** Abnormal flower in one plantlet with multiplied sepals formed at the incorrect position. **(C)** Flowering and fruiting of a runner plantlet (left) and the WT one which was in vegetative growth at June of 2014. Arrows showed the flower and fruit of the runner plantlet, and the stolon of the WT one.

**FIGURE 9:**
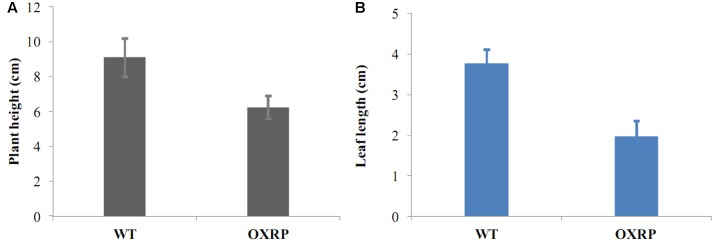
**Runner transgenic plants were dwarf (A)** with smaller leaves **(B)** than the normal ones.

Early-flowering habit was also maintained in the runner plants originated from L1 and L2. And with the continuous asexual propagation, the clones showed a more strong early-flowering phenotype: the third-generation runner clones came into flowering at a very young stage (**Figure 8A**). And unlike the almost unchanged vegetative growth habits of the transformants in the first cropping season, runner clones showed an obviously reduction in vegetative growth. For example, plant showed a dwarf phenotype and their leaf were also extremely smaller than those of the WT ones (**Figure 9**).

Flowering of the runner clones were not only early but also continuously. Some runner plantlets of the transformants even could develop into flower in the hot summer, when the WT ones were still in their vegetative growth phase and only produced stolons (**Figure 8C**). In addition, variation of flower organs was also found in individual plantlets of the progeny, such as the multiplicated sepals (**Figure 8B**) in one runner plantlet originated from L1.

### Gene Expression Detection in the Transgenic Asexual Progeny

To investigate whether the effect of ectopic expression of the transgene could be maintained in their asexual propagule, expression of *EjLFY-1* was also detected in the runner plants originated from the transformants.

**FIGURE 10:**
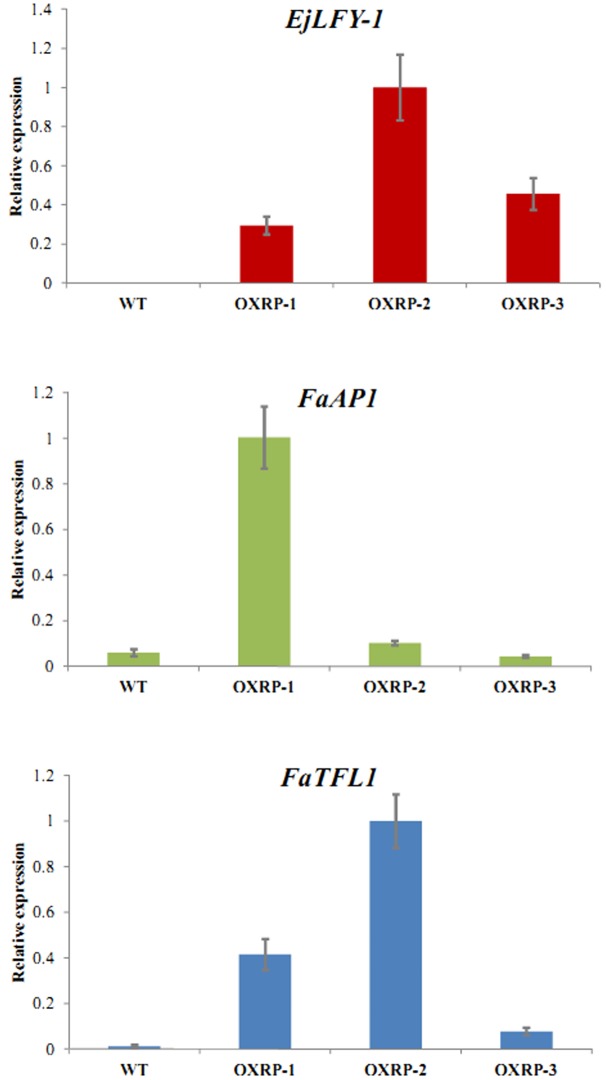
**Expression detection of *EjLFY-1*, *FaAP1*, and *FaTFL1* in third-generation runner plants originated from transgenic strawberry**.

qRT-PCR detection showed that expression of *EjLFY-1* also existed in the third-generation runner plants originated from the transformants, although the expression levels varies (**Figure 10**). As the downstream target gene of *LFY* homologs, expression of *FaAP1*, the *AP1* homolog in strawberry, was also detected. Results showed that although the *EjLFY-1* expression were higher in all the transgenic runner plants than that of the WT ones (in which were zero), expression of *FaAP1* in those plants differed: its expression in OXRP-1 (*Over-expression runner plant 1*) was higher than not only the WT ones but also the OXRP-2 and OXRP-3 (**Figure 10**). The varied expression levels were also detected in *FaTFL1*, the main repressor in flowering process (**Figure 10**). Generally, the *FaTFL1* expression in WT was lower than those in all the three tested transgenic runner plants.

## Discussion

Although the crop improvement of loquat is being carried out both by breeding programs and selection accessions from germplasm resources, the varieties grown mostly come from selections (Badenes et al., 2009). One reason for the slow breeding program in loquat is the existing of its long juvenile phase that last for several years. Genetic engineering technique provides a possible faster breeding method, but it is unavailable for loquat until now for its unclear molecular regulation mechanism of flowering and infeasible transformation system. Compared with other popular fruits, such as apple or citrus, whose genome had been sequenced, molecular mechanism of flowering in loquat is poorly understood and few flowering-related genes have been isolated and characterized.

*LEAFY*-like gene exists not only in angiosperms but also in plant species which don’t produce flowers (Moyroud et al., 2010). As a kind of meristem identity genes related to flowering in seed plants, LFY and its homologs have been demonstrated to not only distribute widely in plants but also function in distantly related species (Peña and Séguin, 2001; Moyroud et al., 2010). One possible reason is that LFY and its homologs are highly evolutionally conserved throughout the plant kingdom and show no apparent similarity to other proteins (Maizel et al., 2005). Two conserved domains of LFY protein were distinguished and, respectively, named as the DBD domain (DNA Binding Domain), which located in the N-terminal and leads it bind to the semi-palindromic 19-bp DNA *cis*-elements of the genes regulated by itself (Sayou et al., 2014), and the SAM domain (Sterile Alpha Motif), which located in the C-terminal and determines the genomic binding landscape (Sayou et al., 2016). Such characteristic motifs were also found in *EjLFY-1* (**Figure 3**), suggesting its possible flowering regulator role in the flowering process of loquat.

Expression analysis revealed that *EjLFY-1* started to increase its expression level in the buds following the transition from vegetative bud to inflorescence bud, decreased its expression level before the forming of flowers and eventually vanished after flowering (**Figure 4**). Comparing with expression of *EjAP1*, the *AP1* homolog of loquat, *EjLFY-1* started and ended its expression earlier (Liu et al., 2013), suggesting its possible key role in the regulation of flowering process of loquat.

To verify the function of *EjLFY-1*, a chimeric 35S::*EjLFY-1* construct was introduced into strawberry. Most of the transgenic plantlets from lines 1 and 2 showed an early flowering phenotype in comparison with the wild-type ones (**Figures 6, 7**). Unlike the reduced vegetative development phenotype in the other reported transgenic plant species over-expressing *LFY*-like genes, strawberry over-expressing *EjLFY-1* didn’t show obvious changes in the vegetative development habit at the first cropping season. Meanwhile the flowers and fruits of the transformants were almost as same as the wild ones (**Figure 6D**). Such results suggest the potential value of *EjLFY-1* in genetic engineering for the fast breeding of loquat. For plantlets belonging to line 3, although the integration and expression were also verified by PCR, they didn’t show the similar early-flowering phenotype as the other two transformants lines. One possible reason for that is the insertion site effect of the transgene. However, it requires further examination.

The long juvenile phase of fruit trees limits their genetic improvement and prevents full domestication of them (Peña and Séguin, 2001). One of the major goals of fruits breeding programs is to reduce their juvenile phase and accelerate floral production. During the last decades, genetic engineering methods based on the use of transgenes have been successfully adopted to reduce the juvenile phase and accelerate the flowering process of some fruit species (Rai and Shekhawat, 2014). However, few reports mentioned the effectiveness and stabilization of the transgene over long periods or its asexual progeny, which was crucial for fruit trees because most of which are perennial and whose propagation in production are usually carried out through asexual reproduction ways in order to ensure reliability.

In this study, the observation results about the transgenic asexual clones lasting for three cropping seasons showed that the early-flowering habit induced by over-expression of *EjLFY-1* could be maintained. Interestingly, with the continuous asexual propagation, the transgenic clones showed even a more consolidated early-flowering trait compared with their ancestor transformants of the first cropping season, such as the reduced vegetative growth habit and even abnormal floral organs (**Figure 8**). Reduction of the vegetative growth traits in those clones maybe caused by the continuous shorten of the vegetative growth, such as the earlier break of dormancy caused by accelerated flowering process. Such finding provides us new insights into the utilization potential of genetic engineering in the breeding of fruits.

The existing of abnormal floral organs in their clones suggests that continuously over-expression of *EjLFY-1* may result in an excessive expression of the downstream floral organ related genes in strawberry, such as the *AP1* homolog which may also control the outer two whorls of floral organs in strawberry just like what *AP1* does in *Arabidopsis* (Bowman et al., 1993). Actually, the expression detection of *EjLFY-1*, *FaAP1*, and *FaTFL1* (**Figure 10**) suggested that the ectopic expression of *EjLFY-1* still exists in those runner clones, following a varied *FaAP1* expression changes compared with the WT ones. TFL1 is a floral repressor which also binds to FD and suppresses the expression of floral promoters *LEAFY* and *AP1* (Hanano and Goto, 2011). With the continuous expression of *EjLFY-1* in strawberry and thereby the high expression level of *FaAP1*, expression of *FaTFL1* was also activated in over-expression runner plants (OXRP) 1 and 2 (**Figure 10**), except the plant OXRP-3 in which the *FaAP1* expression was not at a high level although the *EjLFY-1* was also detected.

Although *LFY* has been demonstrated to function in distantly related species (Peña and Séguin, 2001; Moyroud et al., 2010), this is not the case for all the reported ones. It was shown that over-expression of *AFL1* or *AFL2*, the *LFY* homologs in apple, can induce the early-flowering in *Arabidopsis* (Wada et al., 2002). In other reports, over-expression of *AtLFY* in apple didn’t show the same results instead of a columnar phenotype with shorten internodes (Flachowsky et al., 2010). Similar results were also reported in poplar (Rottmann et al., 2000). Perhaps it was caused by the divergent sequences of the *LFY*-like genes and their interacting factors among different species. In this study, we confirmed that the early-flowering habit existed in strawberry over-expressing *EjLFY-1* and such habit could be maintained in their clones. For the closely evolutional relationship between strawberry and loquat, both belongs to Rosaceae family, it is promising that over-expression of *EjLFY-1* in loquat could also accelerate its flowering process and break its juvenile stage.

## Author Contributions

ZZ and SL conceived and designed research. YL, QZ, NM, HS, and CL conducted experiments. GH and JW contributed plant materials. YL and QZ analyzed data. YL wrote the manuscript. All authors read and approved the manuscript.

## Conflict of Interest Statement

The authors declare that the research was conducted in the absence of any commercial or financial relationships that could be construed as a potential conflict of interest.
