# Supplementary material for: Over-expression of EjLFY-1 Leads to an Early Flowering Habit in Strawberry (Fragaria × ananassa) and Its Asexual Progeny
Source: Front Plant Sci. 2017 Apr 10;8:496. doi: 10.3389/fpls.2017.00496 (PMC5385365; doi:10.3389/fpls.2017.00496)
Supplement: Supplementary file 1 [file Image_1.PDF]

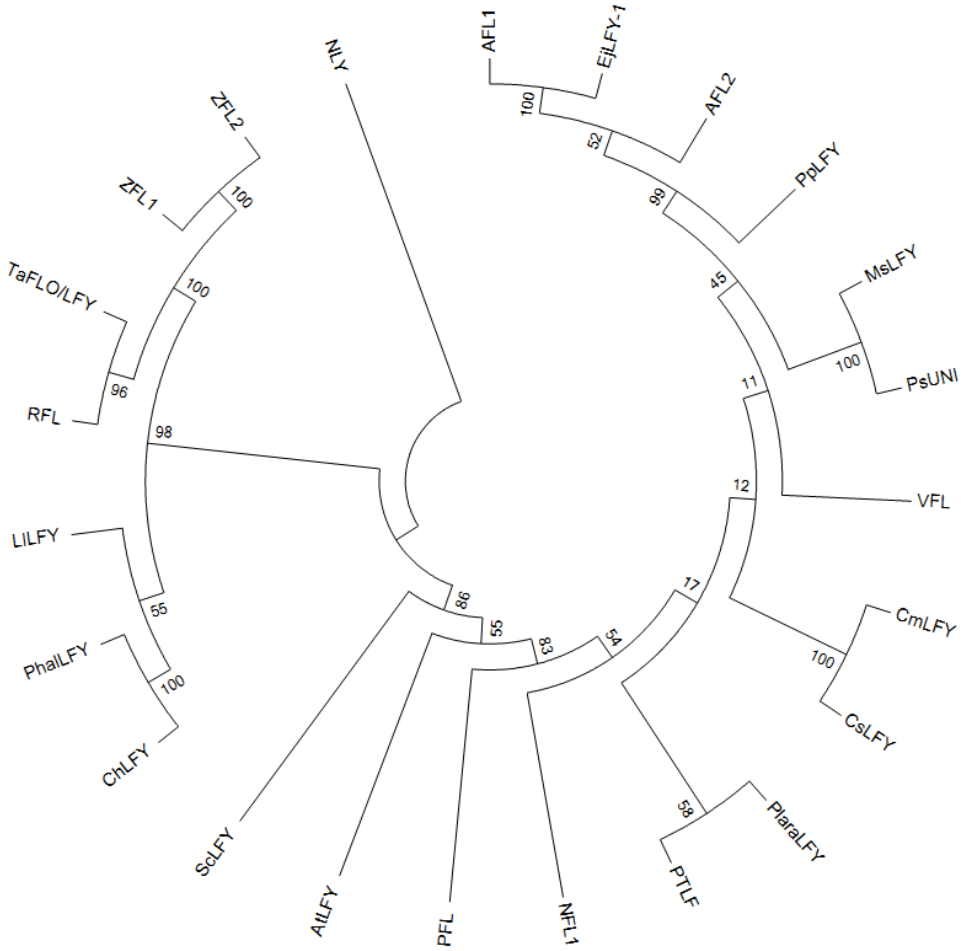

Fig. S1 Phylogenetic tree of the predicted LFY homologues proteins. AFL1, AFL2: *Malus × domestica*, BAB83096, BAB83097; PpLFY: *Pyrus pyrifolia*, ABM63321; MsLFY: *Medicago sativa*, AEO16611; PsUNI: *Pisum sativum*, AAC49782; VFL: *Vitis vinifera*, ADE58105; CmLFY: *Citrus maxima*, ABJ97282; CsLFY: *Citrus sinensis*, AAR01229; PlaraLFY: *Platanus racemosa*, AF106842; PTLF: *Populus balsamifera*, U93196; NFL1: *Nicotiana tabacum*, AAC48985; PFL: *Carica papaya*, DQ054794; AtLFY: *Arabidopsis thaliana*, AAM27941; ScLFY: *Silene coeli-rosa*, AJ311804; ChLFY: *Cymbidium hybrid cultivar*, AGE45851; PhalLFY: *Phalaenopsis hybrid*, FJ469985; LlLFY: *Lilium longiflorum*; ABR13015; RFL: *Oryza sativa*, BAA21547; TaFLO/LFY: *Triticum aestivum*, BAE78665; ZFL1, ZFL2: *Zea mays*, AAO43175, AY179881; NLY: *Pinus radiate*, U76757. The tree was constructed using the Molecular Evolutionary Genetics Analysis version 5 (MEGA5) program (Tamura et al. 2011) with a maximum likelihood method. To estimate the reliability of the tree, a bootstrap analysis was performed with 1,000 replicates.

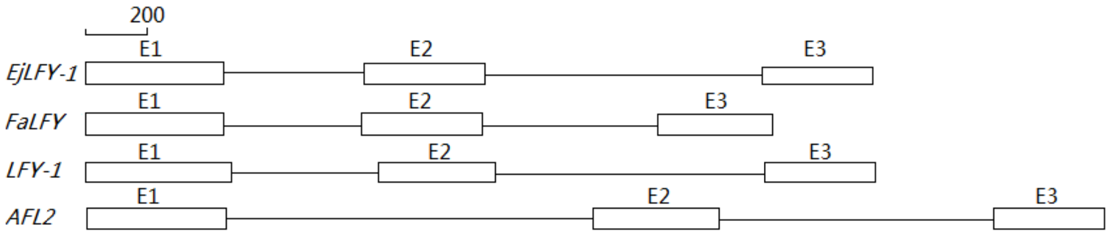

Fig. S2 Schematic diagram of the exons (boxes) and introns (lines) of *EjLFY-1* and other *LFY* homologues. *FaLFY* (JN788261), *LFY* homologue of *Fragaria* × *ananassa*; *LFY-1* (DQ448809), *LFY* homologue of *Glycine Max*; *AFL2* (DQ535886), *LFY* homologue of *Malus* × *domestica*.

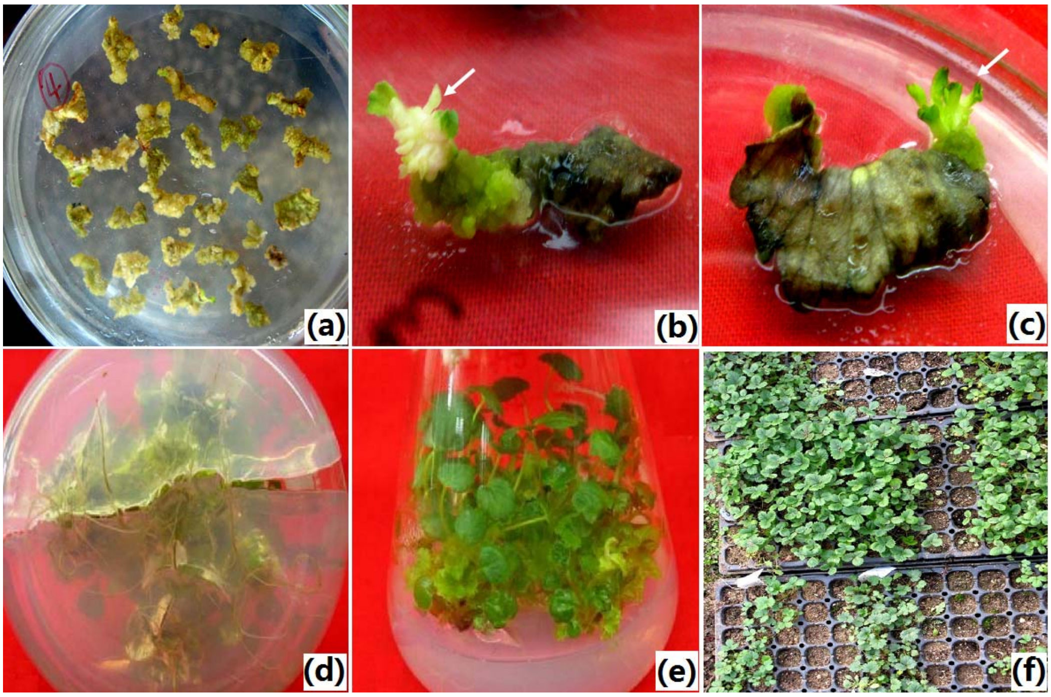

Fig. S3 Generation of transgenic strawberry plants overexpressing *EjLFY-1*. (a) Screening after infestation. (b) Escape bud (*white arrowed*). (c) Resistant bud (*white arrowed*). (d) Rooting cultivation. (e) Proliferating cultivation. (f) Transgenic plants grown in trays.
